# Accuracy and Inter observer variability of blood flow quantification on 4D flow MRI in adult with transposition of the great arteries corrected by arterial switch

**DOI:** 10.1186/1532-429X-18-S1-P153

**Published:** 2016-01-27

**Authors:** Zahra Belhajer, Gilles Soulat, Arshid Azarine, Florence Pontnau, Magalie Ladouceur, Damien Bonnet, Laurence Iserin, Elie Mousseaux

**Affiliations:** 1 grid.10992.330000000121880914Paris Descartes University, Paris, France; 2 grid.414093.bEuropean Hospital Georges Pompidou, Paris, France; 3 grid.412134.10000000405939113Necker hospital for sick children, Paris, France

## Background

4D flow magnetic resonance imaging appears as a reliable tool for blood flow quantification. However, in patients with transposition of the great arteries corrected by arterial switch, the choice of a high velocity encoding (venc) to avoid velocity aliasing due to pulmonary stenosis, could decrease the accuracy of blood flow quantification in vessels such as superior or inferior vena cava (SVC, IVC) and atrio-ventricular valve (AVV) when blood velocities are lower.

Moreover, such accuracy of blood flow estimates can further be influenced by user experience in cardiac imaging due to manual intervention for 3D segmentation process of cardiac structures and correction of background phase offset.

Our aim was to investigate the accuracy and inter observer variability of quantitative MR 4D flow estimates in patients with transposition of the great arteries corrected by arterial switch (asTGA).

## Methods

19 consecutives adults with asTGA underwent a nearly 10 minutes 4D flow MR acquisition (venc ranging from 300 to 400 cm/s, mean 350 in each direction). Two observers with level 1 and 3 in cardiac imaging performed manual correction of background phase offset and flow measurement in SVC, IVC, ascending and descending aorta (AA,DA); pulmonary trunk (PT), mitral and tricuspid valve (MV, TV). Conservation of flow principle was used as an internal physiologic control for comparing blood flow estimates within great arteries (PT vs AA), AVV (TV vs MV), and comparing arterial and venous blood flow of upper body ( AA-DA vs SVC) and lower body ( IVC vs DA) circulations.

## Results

Arterial and venous blood flow measurement (l/min) were better correlated for great arteries (GA) (r = 0,92) or atrioventricular valves (AVV)(r = 0,91) than the upper (r = 0,74) and lower body circulation (r= 0,75). All these correlations were better when blood flow estimates of the level 3 observer were taken into account compared to level 1 observer estimates: GA (r = 0.92; p < 0.0001 vs r = 0.78; p < 0.0001), AVV ( r= 0.91 p < 0.0001 vs r = 0.69; p = 0.008), upper body ( r= 0.74; p = 0.0004; NS) and lower body ( r= 0.75 p = 0.0002 vs r = 0.49; p = 0.03). Bland and Altman analysis further showed smaller mean differences between GA, AVV, upper and lower body when using level 3 observer estimates (0.01; 0.15; 0.25; 0;14 l/min respectively) than when using level 1 observer estimates (0.16; 0.19; 0.27; 0.53 l/min respectively).

When blood flow estimates of the two observers are directly compared, coefficient of variation were 8.7% and 8.1% in AA and PT, 12.5% in DA, 13.5% and 25% in TV and MV, 17.9% and 23.0% in SVC and IVC.

## Conclusions

Taking into account the conservation of flow as an internal validation, accurate blow flow estimates, have been obtained in GA and AV valve of patients with asTGA after using a 350 cm/sec of encoding flow velocity due to pulmonary stenosis. Reliability of 4D flow measurement in venous circulation seems to be lower due to much lower velocities. Furthermore, this accuracy is influenced by observer experience.

